# Tubulin-binding cofactor E-like (TBCEL), the protein product of the *mulet* gene, is required in the germline for the regulation of inter-flagellar microtubule dynamics during spermatid individualization

**DOI:** 10.1242/bio.049080

**Published:** 2020-02-26

**Authors:** James J. Fabrizio, Janet Rollins, Christopher W. Bazinet, Stephanie Wegener, Iryna Koziy, Rachel Daniel, Vincent Lombardo, Dwaine Pryce, Kavita Bharrat, Elissa Innabi, Marielle Villanobos, Gabriela Mendoza, Elisa Ferrara, Stephanie Rodway, Matthew Vicioso, Victoria Siracusa, Erin Dailey, Justin Pronovost, Simon Innabi, Vrutant Patel, Nicole DeSouza, Danielle Quaranto, Amir Niknejad

**Affiliations:** 1 Division of Natural Sciences, College of Mt St Vincent, Bronx, NY 10471, USA; 2 Dept of Biological Sciences St John's University, New York City, NY 11439, USA; 3 Leibniz Institute for Neurobiology Magdeburg, Department Genetics of Learning and Memory, 39118 Magdeburg, Germany; 4 Department of Mathematics, College of Mt St Vincent, Bronx, NY 10471, USA

**Keywords:** *mulet*, TBCE-like, Individualization, Spermatogenesis

## Abstract

Individual sperm cells are resolved from a syncytium during late step of spermiogenesis known as individualization, which is accomplished by an Individualization Complex (IC) composed of 64 investment cones. *mulet* encodes Tubulin-binding cofactor E-like (TBCEL), suggesting a role for microtubule dynamics in individualization. Indeed, a population of ∼100 cytoplasmic microtubules fails to disappear in *mulet* mutant testes during spermatogenesis. This persistence, detected using epi-fluorescence and electron microscopy, suggests that removal of these microtubules by TBCEL is a prerequisite for individualization. Immunofluorescence reveals TBCEL expression in elongated spermatid cysts. In addition, testes from *mulet* mutant males were rescued to wild type using *tubulin-Gal4* to drive TBCEL expression, indicating that the mutant phenotype is caused by the lack of TBCEL. Finally, RNAi driven by *bam*-GAL4 successfully phenocopied *mulet*, confirming that *mulet* is required in the germline for individualization. We propose a model in which the cytoplasmic microtubules serve as alternate tracks for investment cones in *mulet* mutant testes.

This article has an associated First Person interview with the first author of the paper.

## Abstract (precis)

**Summary:** The leading cause of human male infertility is a failure to resolve spermatids from a germline syncytium during spermatid individualization. Here we characterize a mutation that disrupts this process.

## INTRODUCTION

Sperm are matured in a common cytoplasm of germline nuclei, and resolution of individual sperm cells from this syncytium occurs post-meiotically (Roosen-Runge, 1977) in a late step of spermiogenesis known as spermatid individualization. Since individualization defects are the most common cause of human male infertility (Cooper, 2005), and since the giant sperm and convenient genetics of *Drosophila* make the fly an excellent model system in which to study spermatogenesis, we seek to uncover the mechanisms of spermatid individualization using *Drosophila*. Spermatogenesis in *Drosophila* occurs within a germline syncytium, or cyst, that is encased by two somatic cyst cells. As the spermatids grow to a length of 1.8 mm, the cyst stretches to accommodate the flagella. Individualization begins as a membrane-cytoskeleton individualization complex (IC) assembles around each bundle of 64 haploid elongated spermatid nuclei. Each IC is composed of 64 F-actin based investment cones that travel as a coordinated ensemble down the flagella, and each spermatid is individualized by a single cone. As the IC progresses, cytoplasm is removed from between the flagella and membrane is remodeled around each spermatid, forming individualized spermatozoa (Tokuyasu et al., 1972).

In *mulet* (*mlt*) mutant testes, clustering of investment cones is lost and individualization fails, indicating a role for *mlt* in individualization (Fabrizio et al., 1998). Molecular mapping and complementation analyses have identified *mlt* as *CG12214* (Fabrizio et al., 2012; Nuwal et al., 2012), which encodes the *Drosophila* tubulin-binding cofactor E-like (TBCEL) homolog (Celniker et al., 2002). TBCEL is expressed at high levels in testes (Bartolini et al., 2005), consistent with its role in individualization. Unlike its paralog Tubulin-binding cofactor E (TBCE), which participates in microtubule biogenesis (Keller and Lauring, 2005), TBCEL is restricted to microtubule destruction (Bartolini et al., 2005). Rather than binding to intact microtubules, TBCEL disrupts tubulin heterodimers, rendering them unable to polymerize and targeting them for proteasomal degradation (Bartolini et al., 2005; Sellin et al., 2008). The individualization phenotype of *mulet*, together with its preferential testis expression, suggests a role for microtubule disassembly in individualization. Indeed, prior to spermatid nuclear elongation, ∼100 cytoplasmic microtubules form parallel to each spermatid and remain associated with the spermatid throughout elongation (Lindsley and Tokuyasu, 1980). As the nuclei complete elongation, the microtubules remain associated with the flagella but are removed from the vicinity of the nuclei as the IC is assembled. These microtubules disappear at the onset of individualization (Noguchi and Miller, 2003). TBCEL is necessary for the disappearance of these microtubules, and this is a necessary pre-requisite for the coordinated departure of the IC from the nuclear bundle (Fabrizio et al., 2012). Individualization may thus be dependent upon the microtubule architecture of the cyst, and the *mlt* phenotype is likely the result of a defect in the network of cytoplasmic microtubules rather than the IC itself.

Here, we further characterize the *mulet* mutant phenotype, including evidence that the investment cones are functional and confirming that cytoplasmic microtubules persist in *mulet* mutant testes using electron microscopy. We also confirm the localization of TBCEL to elongated cysts, as previously reported (Nuwal et al., 2012), and show using both rescue and RNAi experiments that TBCEL is required in the germline for individualization. We present a model in which the abnormal persistence of cytoplasmic microtubules in *mulet* mutant testes provides alternate ‘tracks’ for investment cones to follow, thus explaining the phenotypes of the various *mulet* mutant alleles and the different knockdown conditions.

## RESULTS

### The investment cones of *mulet* mutant testes exhibit function comparable to wild type

**Fig. 1.**
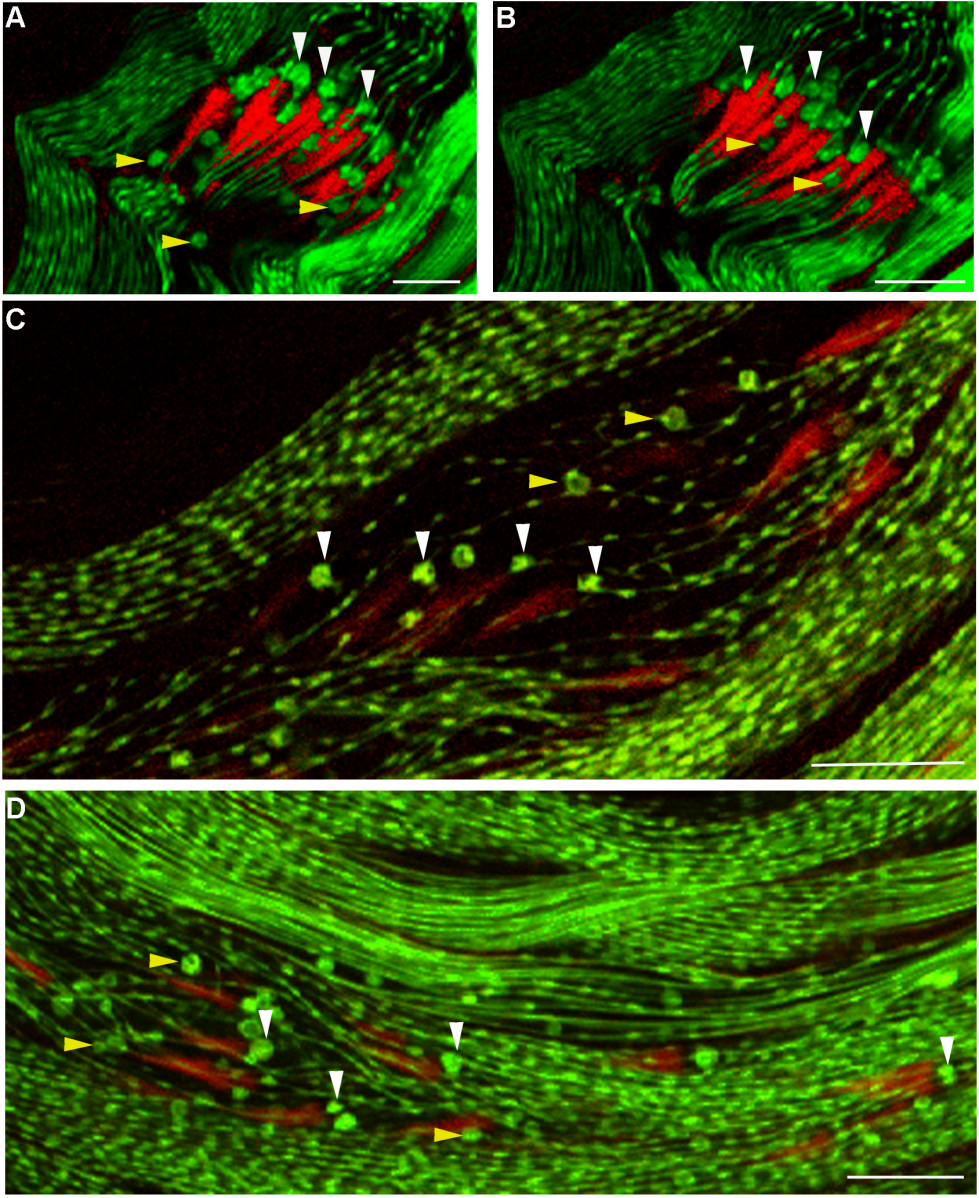
**Mitochondrial dynamics in *mulet* mutant testes as revealed by *don juan-GFP* (*dj-GFP*).** Positive control testes [A,B, *ms(2)4210/CyO; dj-GFP*] depict the coordination between the organized bundle of F-actin cones and a wave front of mitochondrial whorls as revealed by *dj-GFP* (arrowheads). Optical sections through *ms(2)4210/ ms(2)4210; dj-GFP* mutant testes (C,D) reveal asynchronous arrangement of the investment cones that maintain association with mitochondria (white arrowheads). Other mitochondria in both control and mutant testes appear behind the investment cones (yellow arrowheads*)*. Scale bars: 10 µm.

ICs from *mulet* mutant testes fail to individualize spermatids, suggesting either that the investment cones are defective or that the cones are only capable of individualizing spermatids as part of an intact IC. To address this question, *don juan-GFP*, a marker for spermatid mitochondria (Santel et al., 1997, 1998), was crossed into the *mulet* mutant background. In wild-type testes, GFP^+^ mitochondrial whorls are observed ahead of the IC (Bazinet and Rollins, 2003), suggesting that the IC is pushing these mitochondria down the flagella. To see if discoordinated ICs from *mulet* mutant testes are be capable of translocating mitochondria, testes from homozygous *mulet* mutant males and balancer controls, each expressing the *dj-GFP* reporter, were analyzed by confocal microscopy. As expected, most GFP^+^ mitochondria are seen at the base of the investment cones in control testes, suggesting that the cones are pushing GFP^+^ mitochondrial whorls along the flagella (Fig. 1A,B, white arrowheads). Mitochondrial whorls are also commonly seen at the apical tips of investment cones, or ‘behind’ wild-type ICs (Fig. 1A,B, yellow arrowheads). While *mulet* mutant testes reveal an asynchronous arrangement of the F-actin cones and associated GFP^+^ mitochondrial whorls (Fig. 1C,D, white arrowheads), individual Z sections reveal mitochondria associated with the base of the investment cones, suggesting that, as in wild type, individual investment cones are capable of pushing mitochondria along the flagella during spermatid individualization. Also as in wild type, GFP^+^ mitochondria are commonly seen behind mutant investment cones (Fig. 1C,D, yellow arrowheads). Thus, the association between investment cones and mitochondria in *mulet* testes is comparable to wild type. Thus, at least with regard to their ability to translocate mitochondria, investment cones appear to be functional in *mulet* mutant testes, suggesting that the observed individualization failure is due to the discoordination of the IC.

### Ultrastructural analysis of the *mulet* individualization phenotype

**Fig. 2.**
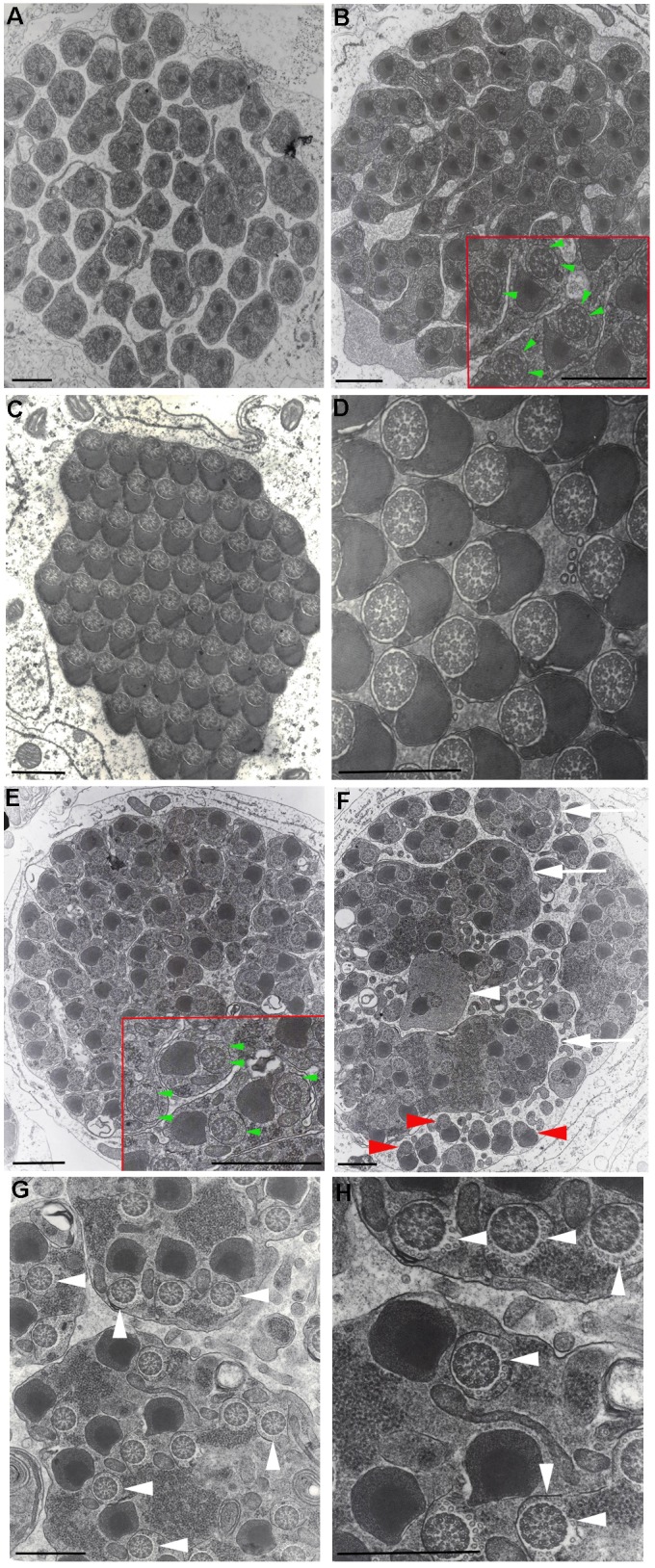
**Cytoplasmic microtubules are in excess prior to spermatid individualization and persist after individualization failure in *mulet* mutant cysts as observed by transmission electron microscopy.** Cross-sections through wild-type (A–D) and *mlt^1^/mlt^1^* (E–H) spermatid cysts are shown at low (12,000×, A,B,E.F), medium (20,000×, C,G) and high (50,000×, D,H) magnifications. Prior to individualization, wild-type cysts exhibit loosely packed sperm tails in a syncytium (A,B). Small, barely distinct cytoplasmic microtubules are observed around the axonemes in one cyst (B, inset, green arrowheads). Cross-sections through wild-type cysts after individualization reveal tightly packed individualized spermatids with little cytoplasm between the flagella (C,D). Cross sections through *mlt^1^* mutant cysts prior to individualization are comparable to wild type (E), but upon close inspection, they reveal an excess of cytoplasmic microtubules around the axonemes (E, inset, green arrowheads). After the passage of the IC, *mlt* cysts reveal large deposits of excess cytoplasm (F, white arrows) as well as the occasional cross-section through an investment cone (F, white arrowheads) and individualized sections of flagella (F, red arrowheads). Axonemes in *mlt* mutant testes are consistently surrounded by an array of cytoplasmic microtubules (G,H arrowheads). Scale bar: 1 µm.

During *Drosophila* spermatogenesis, ∼100 cytoplasmic microtubules are present in elongated spermatid cysts; these microtubules are removed prior to individualization in wild-type testes (Noguchi and Miller, 2003). Previous analyses of *mulet* mutant testes consistently revealed not only disrupted ICs, but the persistence of these microtubules during individualization (Fabrizio et al., 2012). We wondered if this defect would be discernible at the ultrastructural level. To address this question, testes from wild type and homozygous *mlt^1^* mutant males were processed for electron microscopy. Cross-sections through wild-type cysts prior to individualization (Fig. 2A,B) revealed loosely packed spermatids in a syncytium, as previously reported (Tokuyasu et al., 1972). Interestingly, while not discernible in the cyst depicted in Fig. 2A, rings of microtubules are present around the axonemes in the cyst depicted in Fig. 2B (green arrowheads, inset). When they appear, these microtubules are not well defined, suggesting they may be undergoing degradation. After individualization, most of the syncytial cytoplasm is removed and each spermatid is invested in its own plasma membrane, as previously reported (Tokuyasu et al., 1972) and cytoplasmic microtubules are completely absent (Fig. 2C,D). These results are consistent the degradation of cytoplasmic microtubules in wild-type testes.

Prior to individualization, *mlt^1^* mutant cysts are comparable to wild type, exhibiting syncytial sperm (Fig. 2E). An excess of cytoplasmic microtubules is also observed around each axoneme (Fig. 2E, inset), consistent with a loss of TBCEL. After passage of the IC, large deposits of cytoplasm (Fig. 2F, arrow) are evident, consistent with individualization failure. A cross-section through a single investment cone is also visible (Fig. 2F, white arrowhead); the fact that this cone is isolated is consistent with IC discoordination. Upon closer inspection, axonemes of spermatids that fail to individualize are consistently surrounded by microtubules not present in wild type (Fig. 2G,H), confirming that these microtubules persist in the mutant. In addition, some portions of spermatids from *mulet* mutant testes appear individualized (Fig. 2F, red arrowheads), confirming that investment cones in the *mulet* mutant do indeed retain function. Cytoplasmic microtubules are absent from these portions of the flagella (Fig. 2F, red arrowheads), further suggesting that the removal of these microtubules is a necessary prerequisite for individualization.

### Hypomorphic and amorphic *mulet* mutant testes exhibit counterintuitive phenotypes

**Fig. 3.**
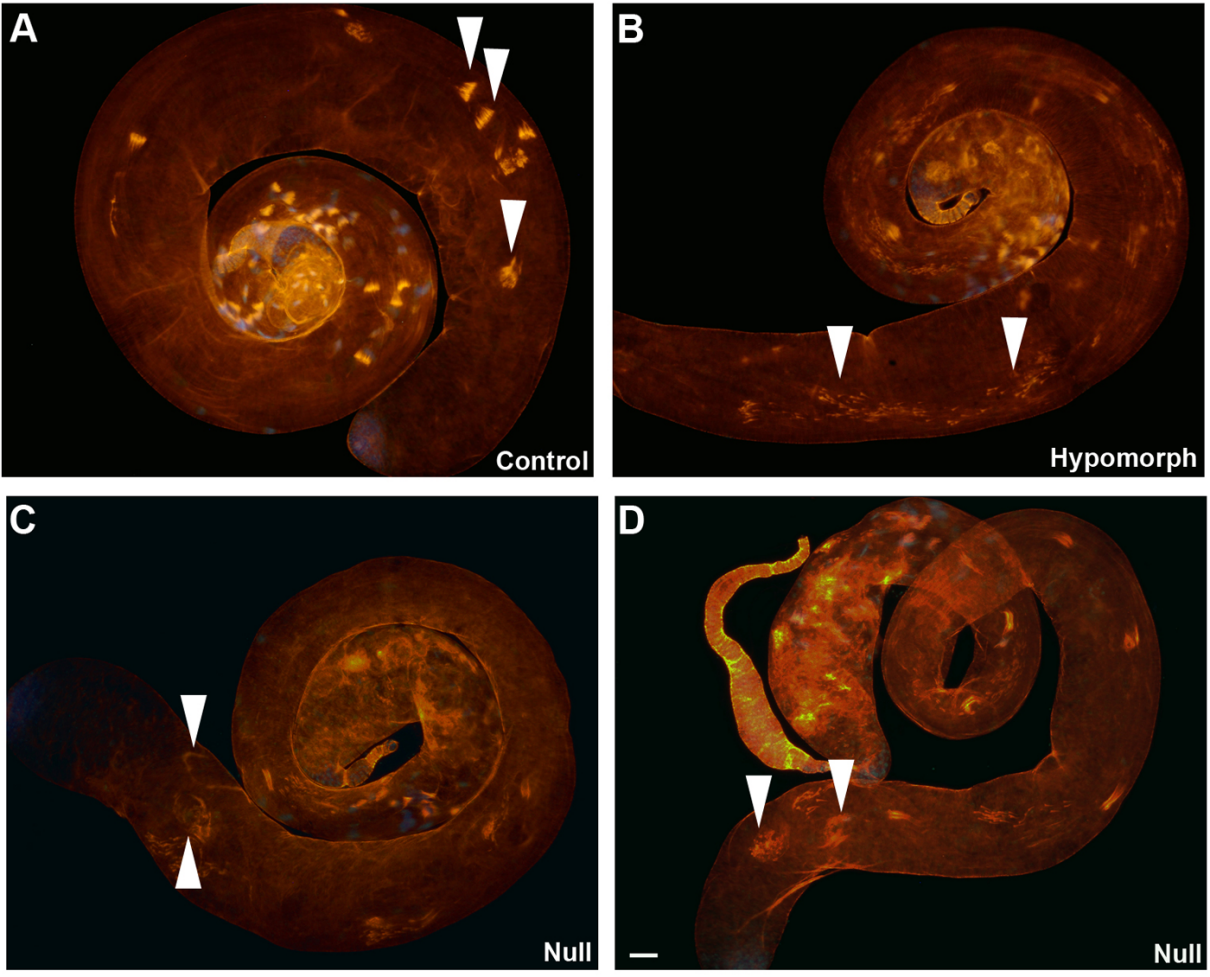
**Hypomorphic and amorphic *mulet* mutant males exhibit counterintuitive individualization phenotypes.** Wild-type testes exhibit intact ICs that form waste-bags at the apical end of the testis (arrowheads in A). While testes from hypomorphic *mlt[EP-CG12214]/Df* mutant males exhibit severely disrupted ICs that fail to form waste bags (arrowheads in B), testes from homozygous null *mlt[G18151]* mutant males exhibit mildly disrupted ICs that appear to form waste-bag-like structures at the apical end of the testes (arrowheads in C,D)*.* Scale bars: 30 µm.

The *mulet* mutation has a distinctive individualization phenotype observable using a fluorescence assay in which the F-actin component of the IC is stained using phalloidin (Fabrizio et al., 1998, 2012). While wild-type testes exhibit intact ICs that ultimately form waste-bags at the apical end of the testis (Fig. 3A, arrowheads), testes from hypomorphic *mlt[EP-CG12214]/Df (2R)BSC281* mutant males exhibit severely disrupted ICs that fail to form waste bags (Fig. 3B, arrowheads). Paradoxically, testes from homozygous null *mlt[G18151]* mutant males exhibit mildly disrupted ICs that form waste-bag-like structures at the apical end of the testes (Fig. 3C,D, arrowheads). Thus, a partial reduction of TBCEL results in a more severe phenotype than in absence of TBCEL.

### TBCEL protein is expressed in elongated spermatid cysts

In an earlier study, TBCEL was localized to elongated cysts, particularly to the syncytial portion apical to the IC (Nuwal et al., 2012). To confirm these results, we prepared testes for immunofluorescence using the same polyclonal anti-TBCEL antibody (Nuwal et al., 2012; see Materials and Methods for generation of antiserum). We confirmed the localization of TBCEL to elongated cysts, though at a primary antibody dilution of 1:50, rather than the reported dilution of 1:1000 (Fig. S1A,B). We were unable to detect any preferential localization ahead of the IC. The specificity of our assay was confirmed by the background level of TBCEL staining observed in hemizygous testes (Fig. S1C,D) indicating that the anti-TBCEL antiserum reliably detects TBCEL.

### Expression of TBCEL in the testis rescues the *mulet* mutant phenotype

In order to determine if the absence of TBCEL is responsible for the *mulet* phenotype, we used the GAL4/UAS system to overexpress TBCEL in the testis. For scoring purposes, testes were characterized as exhibiting ‘complete rescue’ if they were indistinguishable from wild type, and ‘partial rescue’ if IC structure was improved relative to the negative control mutant testes but less organized than wild type. Partial or complete rescue of *mulet* would indicate that the *mulet* mutant phenotype is due to a deficiency of TBCEL.

We searched for GAL4 drivers that would overexpress TBCEL in post-meiotic cysts. Given its high level of expression in the germline through the spermatocyte stage, the most promising candidate was *alphaTub84B-Gal4*, commonly known as *tub-Gal4* (White-Cooper, 2012). Membrane-bound GFP expression under *tub-*Gal4 control was observed in spermatocyte membranes (Fig. S2A,B, arrowheads) and in the membranes of elongated spermatid cysts (Fig. S2A–D, arrows). GFP expression in elongated cysts could be attributed to somatic expression or combined germline and cyst cell expression, but the high level of expression in the germline up to the spermatocyte stage suggests that at least some of the expression was in the germline. To test if *tub-Gal4* could specifically drive TBCEL in the testis, UAS-SMN-TBCEL was placed under *tub-Gal4* control, and testes were dissected from 0–1 day old *tub-Gal4; UAS-SMN-TBCEL* males and processed for immunofluorescence using an anti-SMN antibody. While control testes exhibited background staining (Fig. S2G,H), testes dissected from *tub-Gal4; UAS-SMN-TBCEL* males exhibited anti-SMN staining in a ribbon-like pattern (Fig. S2E,F), consistent with SMN-TBCEL expression in elongated cysts. Taken together, *tub-Gal4* seemed the logical choice for a GAL4 driver capable of effecting rescue of the *mulet* mutant phenotype.

**Fig. 4.**
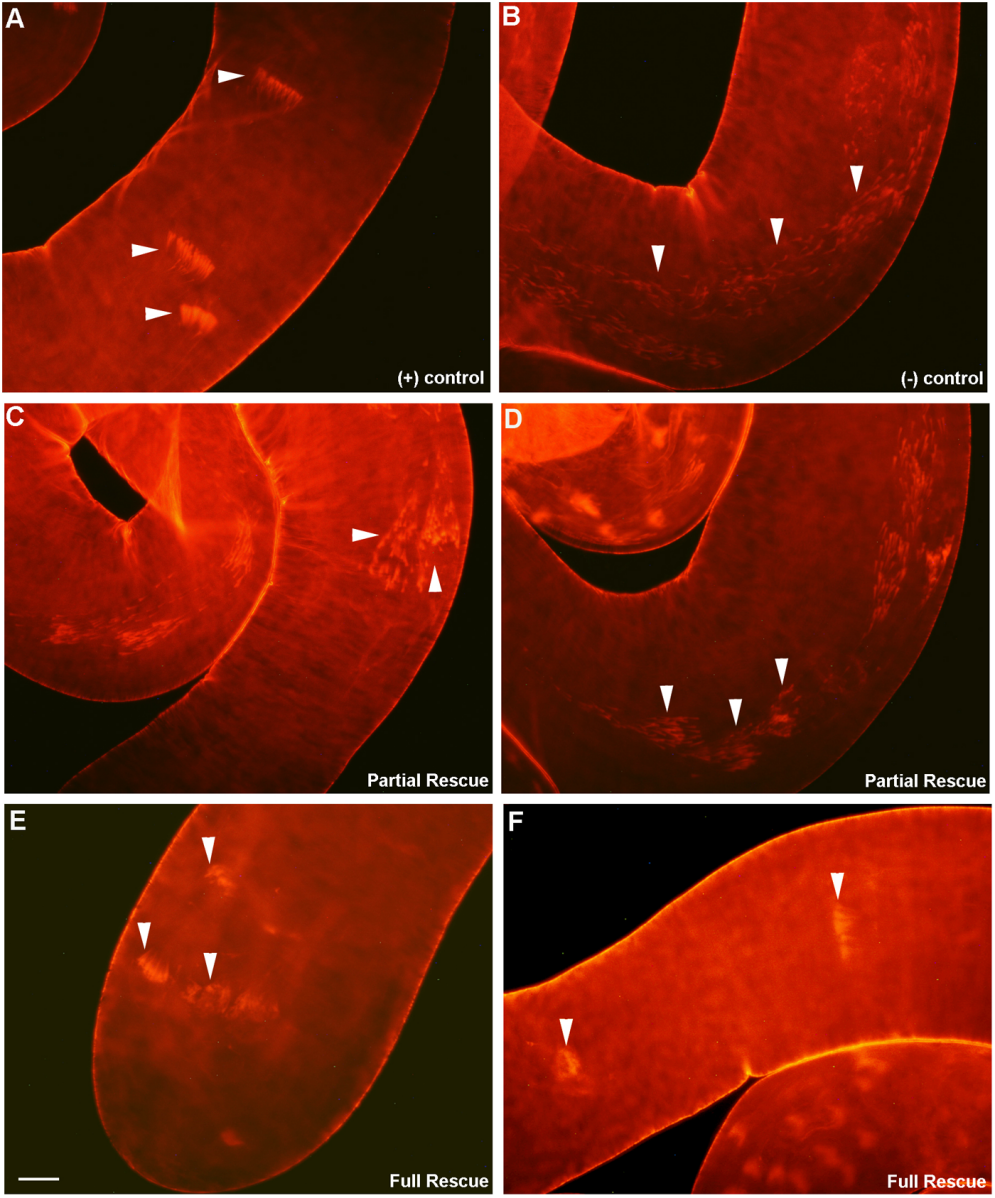
**Rescue of the *mulet* mutant phenotype using *tub-Gal4*.** Positive control testes [A, *EP-CG12214* or *Df(2R)BSC281/CyO*] reveal intact ICs (arrowheads in A), while investment cones in the negative control [B, *EP-CG12214/Df(2R)BSC281*] are disorganized (arrowheads in B). Expression of TBCEL under *tub-Gal4* control in hemizygous mutant testes most often resulted in partial rescue of the phenotype [C,D, *EP-CG12214/Df(2R)BSC281; tub-Gal4 UAS-mCD8-GFP*] as characterized by improved organization of the IC (arrowheads in C,D). Males with two copies of the *EP-CG12214* (E,F; *EP-CG12214/EP-CG12214;tub-Gal4 UAS-mCD8-GFP*) very often exhibited full rescue, as characterized by ICs that were indistinguishable from wild type (arrowheads in E,F)*.* Scale bar: 20 µm.

Rescue was attempted three different ways in a series of nine independent experiments. To effect rescue, the *tub-Gal4* and *UAS-SMN-TBCEL* chromosomes were crossed into a hemizygous *ms(2)4210/Df* or *EP-CG12214* mutant background, or *tub-Gal4* was crossed into a homozygous *EP-CG12214* mutant background. Since *EP-CG12214* is a P{UAS} insertion into the 5′ UTR of *mulet*, this line may be used alone as a hypomorphic mutant allele or to effect rescue when crossed with an appropriate Gal4 driver. Indeed, homozygous *EP-CG12214* males possess two *UAS-TBCEL* chromosomes, so we anticipated complete rescue to wild type in these males after introduction of *tub-Gal4.* Similarly, two rescue constructs are also present in *EP-CG12214/Df; UAS-SMN-TBCEL* males, thus we were confident that we would achieve complete rescue in these males. Finally, *ms(2)4210/Df; UAS-SMN-TBCEL* testes only possess one rescue construct in a hemizygous mutant background, and thus we were not anticipating complete rescue in these males. In all experiments, testes were dissected from 0–1 day old males and processed for staining with Rhodamine Phalloidin. Positive control testes dissected from heterozygous males always appeared wild type (Fig. 4A and Table 1). Negative control testes were dissected from either homozygous or hemizygous mutant males missing either the *tub-Gal4* driver or the rescue construct. While most negative control testes exhibited a strong *mulet* mutant phenotype (Fig. 4B), a few revealed a scattering of investment cones that was somewhat less disorganized, and thus, in the interest of accuracy, were scored as ‘partial rescue’ (Table 1).

**Table 1.**
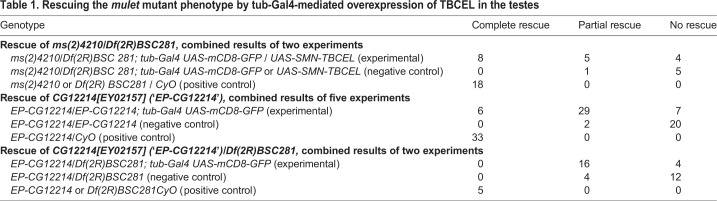
**Rescuing the *mulet* mutant phenotype by tub-Gal4-mediated overexpression of TBCEL in the testes**

Despite the presence of two rescue constructs, expression of UAS-SMN-TBCEL and *EP-CG12214* under *tub-Gal4* control in hemizygous mutant testes resulted in partial rescue of the phenotype (Fig. 4C,D and Table 1) as characterized by improved organization of the F-actin cones; complete rescue was never observed in these testes. Complete rescue was not uncommon with UAS-SMN-TBCEL in *ms(2)410/Df(2R)BSC281* testes (Table 1). Moreover, the experimental group was 78.4% similar to the positive control, and only 48% similar to the negative control using a cosine similarity test, indicating that these data are significant. Complete rescue was more common in males with two copies of *EP-CG12214* (*EP-CG12214/EP-CG12214;tub-Gal4 UAS-mCD8-GFP*; Fig. 4E,F and Table 1). Indeed, when rescue was attempted with two copies of *EP-CG12214*, Euclidian distance measurements indicate that for the three rescue conditions (complete, partial and no rescue, see Table 1), testes from the experimental males, possessing both *tub-Gal4* and *EP-CG12214*, were more similar to the positive control than the negative control was similar to the positive control (40.2% versus 20.9%, respectively), indicating that these data are significant. While partial rescue was achieved with one copy of the *EP-CG12214* in 16/20 males (Table 1), Euclidian distance measurements reveal that the experimental group was nearly as similar to the negative control as the positive control was similar to the negative control (14.4% versus 13.6%, respectively), indicating that these data are not significant, consistent with the prediction that rescue is more efficient with two copies of *EP-CG12214.* Thus, rescue of *mulet* was achieved by driving TBCEL expression in the testis, indicating that the phenotype is a direct result of a TBCEL deficiency.

We wished to see if rescue, as observed using fluorescence microscopy, also restored fertility in rescued males. Unfortunately, the *tub-Gal4; UAS-SMN-TBCEL* stock used in the above experiments was lost. But we were still able to test the ability of the *EP-CG12214* chromosome to restore fertility in standard fertility assays, where individual males were crossed against two *yw* virgin females at 25°C. While hemizygous *EP-CG12214/Df* are completely sterile and *EP-CG12214/CyO* males are fertile, *EP-CG12214/Df; tub-Gal4* males exhibit improved fertility compared to hemizygous mutant males (Table S1). The fact that fertility in the experimental group is weak is consistent with the partially rescued ICs observed in testes from these males. Similar results were obtained when homozygous *EP-CG12214/EP-CG12214* males were rescued using *tub-Gal4* (Table S1). Thus, rescue scored using the fluorescence assay does translate into increased fertility, at least with the *EP-CG12214* chromosome.

### Germline-specific knockdown of TBCEL using *bam-Gal4-VP16* phenocopies the *mulet* mutation

If *mulet* is required in the germline for spermatid individualization, then RNAi-mediated knockdown of TBCEL in the germline should also produce the discoordinated investment cones and persistent inter-flagellar microtubules characteristic of *mulet* mutant testes. Given the inverse relationship between the severities of the *mulet* alleles and the spermatogenic phenotype, we predicted that the observed spermatogenic phenotype may actually become less severe as the level of RNAi increased.

Several GAL4 drivers shown to drive post-meiotic germline expression (White-Cooper, 2012) were used to drive expression of double-stranded *mulet* RNA and tested for their ability to phenocopy the individualization defect of *mulet*. Surprisingly, *tubulin-*GAL4, which was successfully used in our rescue experiments, and *hsp-*GAL4, which drives expression in the post-meiotic germline (White-Cooper, 2012), produced no recognizable spermatogenic phenotype when crossed to *CG12214* RNAi at 25°C. Predictably, *nanos-*GAL4, which drives expression in germline stem cells and their immediate progeny (White-Cooper, 2012) did not produce any observable phenotype. Interestingly, *CG12214* RNAi driven by *bam*-GAL4-VP16, which specifically drives expression in spermatogonia, produced spermatogenic defects similar to what is observed in *mulet* mutant testes, indicating that TBCEL is specifically required in the germline for individualization.

We first set out to determine if TBCEL levels were reduced when *CG12214* RNAi was driven by *bam*-GAL4-VP16. Flies possessing the *bam-GAL4-VP16* driver were crossed to flies carrying UAS-*CG12214* RNAi at 25°C. Testes dissected from the resulting *trans*-heterozygous males were dissected and processed for immunofluorescence using guinea pig anti-TBCEL (Nuwal et al., 2012). When compared to testes dissected from control males possessing UAS-*CG12214* RNAi alone, which exhibited prominent TBCEL expression in elongated cysts (Fig. S3A), testes dissected from knockdown males exhibited background levels of TBCEL (Fig. S3D). Since GAL4 activity is enhanced at elevated temperatures (Duffy, 2002), crosses were performed at 28°C to enhance RNAi knockdown. Indeed, these testes also revealed knockdown of TBCEL (Fig. S3E, compare with B), but we were unable to detect differences in knockdown between the two temperatures. We tried to achieve maximum knockdown of TBCEL by including *UAS-Dicer* along with UAS-*CG12214* RNAi under *bam-GAL4-VP16* at 28°C, and again, we observed substantial knockdown of TBCEL levels in the testis (Fig. S3F, compare with C) that were indistinguishable from levels observed without Dicer at 25°C and 28°C, indicating that our antibody is not sensitive enough to distinguish among low levels of TBCEL expression.

**Fig. 5.**
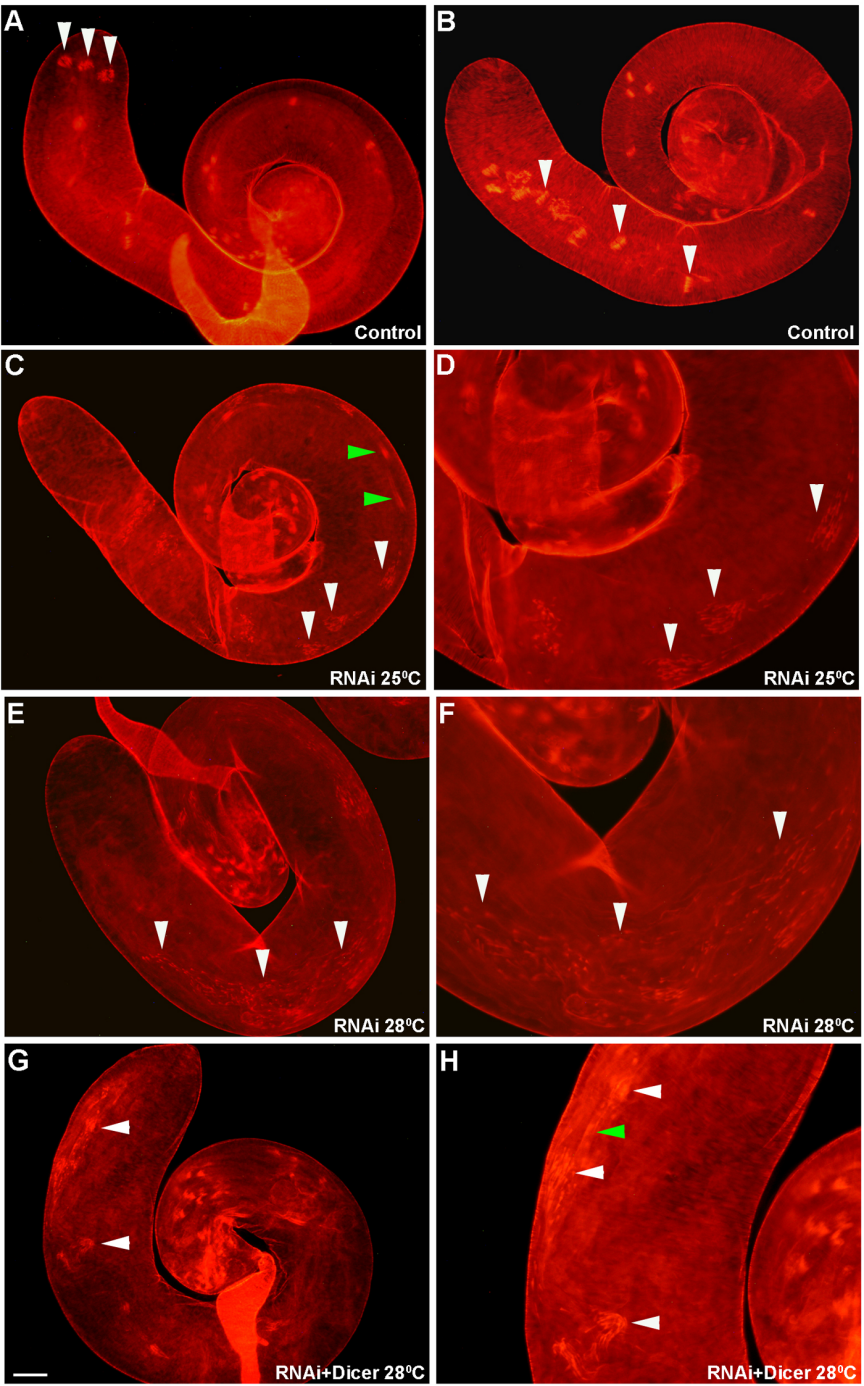
**Germline-specific knockdown of TBCEL using *bam-Gal4-VP16* phenocopies *mulet*.** (A,B) Low magnification (100×) images of testes dissected from negative control *UAS-CG12214 RNAi* males. (C–H) Images on the right are higher-magnification (200×) close-ups of images on the left (100×). Control testes exhibit the presence of waste-bags and intact ICs (arrowheads in A and B, respectively). *UAS-CG12214RNAi; bam-Gal4-VP16* testes exhibited IC defects comparable to *mulet*. Specifically, at 25°C there was mild disruption of the ICs (white arrowheads in C,D)*.* (E,F) More severe disruptions were observed at 28°C (white arrowheads in E,F), consistent with a higher level of RNAi at the elevated temperature. Milder defects, comparable to the null mutant phenotype, were observed in testes dissected from *UAS-CG12214RNAi*/*UAS-dicer; bam-Gal4-VP16* males (white arrowheads in G,H). Green arrowheads in C and H indicate F-actin sleeves, which were regularly observed in testes from knockdown males. Scale bars: (A–C,E,G) 40 µm; (D,F,H) 20 µm.

Control testes dissected from males possessing only the *UAS-CG12214 RNAi* chromosome without the *bam-GAL4-VP16* driver exhibited normal ICs that remain intact until the waste-bag stage (Fig. 5A,B, arrowheads and Table 2)*,* while testes dissected from experimental males expressing *CG12214 RNAi* driven by *bam-GAL4-VP16* reared at 25°C revealed mildly disrupted ICs, as characterized by the close proximity of the scattered investment cones such that the ICs almost looked intact (Fig. 5C,D, arrowheads and Table 2). Disrupted ICs characteristic of hypomorphic mutations in *mulet* in which the scattering was so severe that it was difficult to assign the scattered cones to a particular complex, made up the majority of ICs at 25°C (Table 2). These data were also processed using Chi-Square analysis; a *P*-value of 3.19×10^−37^ was calculated, indicating that there is a significant difference between the experimental and the control. When flies were reared at 28°C to increase GAL4 activity and RNAi (Duffy, 2002), the scattering of investment cones became more severe and indistinguishable from *mulet* hypomorphs (Fig. 5E,F, arrowheads and Table 2). Here as well, Chi-Square analysis revealed an extraordinarily small *P*-value of 2.45×10^−48^, also indicating a significant difference from the negative control. Taken together, RNAi against *CG12214* in the germline successfully phenocopied the individualization defect of *mulet* hypomorphs*,* and stronger RNAi induced at the elevated temperature worsened the individualization phenotype.

**Table 2.**
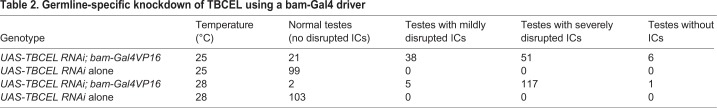
**Germline-specific knockdown of TBCEL using a bam-Gal4 driver**

In order to maximize RNAi, *UAS-Dicer* and *UAS-CG12214 RNAi* were placed under *bam-GAL4-VP16* control at 28°C. We reasoned that enhanced RNAi would result in a severe knockdown of *CG12214* RNA that would phenocopy null mutations in *mulet.* As predicted, testes dissected from these males exhibited more mild disruptions (Table 3). Testes with intermediate disruptions, in which the IC was obviously disrupted but ownership of the investment cones by a particular IC was obvious, were also prominent in these males (Table 3). Indeed, ICs with both mild and intermediate disruptions often formed waste-bag-like structures toward the apical end of the testis (Fig. 5G,H, arrowheads) much like the previously documented null mutant phenotype (Fig. 3C). These results were validated using the Chi-Square test, which revealed significant phenotypic differences between testes undergoing RNAi and the control (*P*=3.51×10^−15^). Thus, enhancement of RNAi in the germline reduced the severity of the observed IC disruptions and successfully phenocopied null mutations of *mulet.*

**Table 3.**
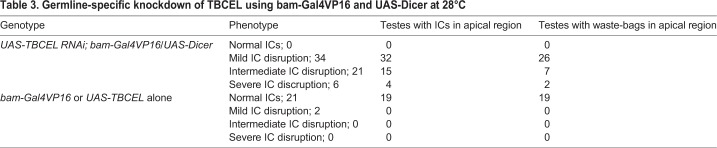
**Germline-specific knockdown of TBCEL using bam-Gal4VP16 and UAS-Dicer at 28°C**

RNAi phenotypes observed by fluorescence microscopy were confirmed by fertility testing of individual males. While control *UAS-CG12214 RNAi* males reared at either 25°C or 28°C were fertile (Table S2), moderate knockdown of *CG12214* at 25°C by *bam-GAL4-VP16* only reduced the fertility of individual males (Table S2). Increasing the level of RNAi by raising the temperature to 28°C or including *UAS-Dicer* at 25°C resulted in almost complete sterility (Table S2). Most notably, further knockdown of TBCEL by including *UAS-Dicer* at 28°C resulted in improved fertility (only 1/9 males was completely sterile, see Table S2), consistent with earlier observations of improved IC structure in null mutant males. RNAi against *CG12214* in the germline therefore phenocopied *mulet*, confirming that *mulet* is required in the germline for individualization.

## DISCUSSION

In this work, we have expanded our knowledge of the role of TBCEL in *Drosophila* spermatid individualization. We further characterized the *mulet* phenotype, and confirmed the spermatogenic defect was the result of a disruption in *mulet* using rescue experiments*.* RNAi experiments revealed a germline-specific requirement for *mulet* and led to a model connecting microtubule dynamics and spermatid individualization (see below). These data underscore the importance of a specific cystic environment as a prerequisite for proper spermatid individualization.

### The *mulet* mutant phenotype, as further revealed by a mitochondrial marker and electron microscopy

*mulet* was originally identified in a P-element mutagenesis screen for male-sterile mutations (Castrillon et al., 1993) and later revealed to be an individualization defect (Fabrizio et al., 1998). In *mulet* mutant testes, investment cones successfully assemble around elongated spermatid nuclei, but become discoordinated upon departure, producing a disrupted IC that fails to individualize spermatids (Fabrizio et al., 1998, 2012). This individualization failure is thus likely due to the discoordination of normal investment cones. Alternatively, the function of the investment cones themselves may be compromised, producing discoordination as a consequence of a failure within each cone. We think this unlikely for three reasons. First, whorls of mitochondria, revealed by *don juan-GFP* and present ahead of the investment cones in wild-type testes (Bazinet and Rollins, 2003) are also observed ahead of investment cones in *mulet* mutant testes. These investment cones, while discoordinated, are thus still capable of pushing mitochondria along the cyst. Secondly, electron microscopy revealed individualized portions of spermatids in *mulet* mutant cysts, indicating that the investment cones are individualizing part of the sperm tail before arrest. Finally, since investment cones are composed of a meshwork at the base consisting of F-actin filaments crosslinked by Arp 2/3, and an apical tail consisting of bundled, parallel F-actin microfilaments crosslinked by villin and fascin (Noguchi et al., 2008), defects in cone structure are easily observed. For example, when Arp 2/3 is absent, the apical meshwork is not formed, and rather than appearing triangular, investment cones appear thin. In contrast, if villin or fascin is absent, the parallel bundles are not formed, and the investment cones appear as widened triangular plows without a tapered apex (Noguchi et al., 2008). Neither of these defects is observed in *mulet* mutant testes; the investment cones are comparable to wild type. Thus, since investment cones in *mulet* mutant testes are normally shaped, capable of translocating mitochondria and partially individualizing spermatid flagella, we favor the idea that the observed defect is due primarily to investment cone discoordination. It is interesting to note that the one investment cone from a *mulet* mutant testis visible in our electron micrographs was structurally abnormal, missing visible membrane around part of its circumference (Fig. 2F). This absence of membrane could be biological, or it could be only apparent and due to oblique sectioning of the cone. Further cross-sectioning through *mulet* mutant investment cones will discriminate between these possibilities.

The underlying cause of investment cone discoordination appears to be the abnormal persistence of cytoplasmic microtubules (Fabrizio et al., 2012), which, prior to individualization, work interdependently with mitochondria to facilitate spermatid elongation (Fabian and Brill, 2012). Normally, these microtubules disappear just prior to individualization (Noguchi and Miller, 2003) and TBCEL, the protein product of *mulet*, appears to be responsible for their removal (Fabrizio et al., 2012). The electron microscopic data presented in this paper, revealing excess cytoplasmic microtubules surrounding the axonemes of spermatids that have failed to individualize in *mulet* mutant cysts, are consistent with this model. Electron microscopy has also revealed these microtubules in a state of degradation in wild-type cysts prior to individualization, consistent with their removal as a necessary prerequisite for the passage of the IC. Since fluorescence microscopic analysis has only been able to detect the presence of these microtubules in elongating cysts and their disappearance just prior to individualization (Noguchi and Miller, 2003; Fabrizio et al., 2012), we believe that these data have uncovered the degradation of cytoplasmic microtubules by TBCEL. Perhaps this step escaped observation via immunofluorescence due to the loss of immunoreactivity during the degradation step of the acetylated α-tubulin marker used to detect cytoplasmic microtubules.

Also interesting is the absence of cytoplasmic microtubules from the individualized portion of spermatids in *mulet* mutant cysts, further confirming that removal of these microtubules is a requirement for individualization. Some clues as to why microtubule persistence might disrupt the IC come from studies revealing crosslinking interactions between these microtubules and between these microtubules and spermatid mitochondria (Fabian and Brill, 2012). These connections, mediated by Milton and dMiro, form a crosslinked network on the mitochondrial surface that both permit and maintain the elongated state of spermatid mitochondria (Noguchi et al., 2011) and could act as a barrier to IC passage. Indeed, in *Cds* mutant males, where Diacylglycerol (DAG) levels are reduced, there is an overgrowth of both spermatid mitochondrial derivatives and endomembranes, leading to discoordinated ICs like those observed in *mulet* mutant testes (Laurinyecz et al., 2016), again suggesting that the failure to remove molecular ‘clutter’ in the cyst can result in individualization failure. The situation may be more complex since *mulet-*like mutant phenotypes may also result from reduced mitochondria and unstable axonemes caused by a reduction in tubulin polyglycylation (Mendes Maia et al., 2014).

### Localization of TBCEL to elongated cysts

TBCEL has already been shown to localize to bundles of elongated spermatids apical to the IC (Nuwal et al., 2012). In contrast, we observed an even distribution of TBCEL throughout elongated cysts. In addition, the reported 1:1000 dilution of polyclonal anti-TBCEL antibody failed to detect TBCEL in our preparations; a 1:50 dilution of antibody was most optimal in our hands. These discrepancies may be explained by differences in microscopy and the degraded quality of the antibody; while our results were obtained using conventional wide-field epi-fluorescence microscopy, confocal microscopy was employed in the earlier study. Moreover, the quality of the antibody may be degraded between the times of our experiments. We must therefore concede that a more precise localization of TBCEL was obtained in the earlier study.

### Expression of TBCEL in the testis rescues the *mulet* mutant phenotype

We employed the *tub-Gal4* driver, which had been previously shown to drive expression through the spermatocyte stage and also in cyst cells through spermatid elongation (White-Cooper, 2012), to express TBCEL in *mulet* mutant backgrounds and rescue individualization. Analysis of membrane-anchored GFP expression under *tub-Gal4* control in live squash preparations reveals GFP^+^ spermatocyte membranes, confirming germline expression. GFP expression was also observed in the membranes of elongated cysts, which may represent expression in elongated cyst cells, as previously reported (White-Cooper, 2012) or expression in the post-meiotic germline. In either case, we thought that *tub-Gal4* might be an appropriate driver to effect rescue. Indeed, *tub-Gal4* successfully drove SMN-tagged TBCEL expression in a pattern consistent with expression in elongated cysts, indicating that the effect was not limited to mCD8-GFP expression.

Rescue was first attempted in hemizygous *mlt^ms(2)4210^* mutant males; *ms(2)4210* is a hypomorphic *P{LacW}* insertion into a region of the 5′ UTR of *mulet* common to all three isoforms of *mulet* mRNA. Thus, placing this allele in *trans* to a deficiency that uncovers *mulet* would generate a strong phenotype. We reasoned that introducing only one copy of *UAS-SMN-TBCEL* under *tub-Gal4* control would not likely result in the complete rescue of the mutant phenotype. Indeed, we were surprised to see nearly one third of males hemizygous for *mlt^ms(2)4210^* exhibit complete rescue when SMN-TBCEL expression was driven by *tub-Gal4*, while nearly half exhibited improved IC structure and were thus scored as ‘partial rescue’.

In contrast, we predicted that males homozygous for the *EP-CG12214* chromosome, and thus possessing two copies of *UAS-TBCEL*, would exhibit more efficient rescue*.* While we did observe partial rescue in most cases, complete rescue was actually less prevalent than *tub-Gal4* driving one copy of *UAS-SMN-TBCEL.* We considered that this discrepancy might be due to differences between the two mutant alleles being rescued. However, given that *mlt^4210^* and *CG12214^EY02157^* are seven bases apart in a region of the 5′ UTR common to all isoforms of the *mulet* transcript, we think this unlikely. Rather, since the insertion of *P{EP}* actually disrupts the 5′ UTR of *mulet*, we reasoned that all overexpressed transcripts would possess disruptions of their 5′ UTRs due to the insertion of the P-element. In contrast, the *UAS-SMN-TBCEL* chromosome, in which the entire TBCEL gene including the 5′ UTR is under UAS control, would produce a transcript with a complete 5′ UTR and not susceptible to translational regulation problems. Interestingly, when both *EP-CG12214* and *UAS-SMN-TBCEL* chromosomes were driven simultaneously in order to rescue hemizygous *EP-CG12214/Df* males, no complete rescue was achieved. Perhaps this is due to interference between the SMN-tagged TBCEL from one chromosome and the unmodified TBCEL produced from the other.

In order to determine if the rescued testes translated into increased fertility, we repeated the rescue experiments to assay the fertility of individual males. Unfortunately, we lost the *tub-Gal4; UAS-SMN-TBCEL* stock used to rescue the *mlt^4210^* mutant males, and the *EP-CG12214* stock stopped producing homozygous mutant flies. Thus, we attempted to rescue hemizygous *EP-CG12214/Df* males simply by introducing the *tub-Gal4* driver. The fertility of these experimental males was superior to that of hemizygous males without the driver and inferior to that of heterozygous males. Thus, rescue by overexpression of TBCEL using *tub-Gal4* is observable both by improved IC structure and male fertility.

### Germline knockdown of TBCEL phenocopies *mulet*

RNAi-mediated knockdown of TBCEL using the germline-specific *bam-Gal4-VP16* driver phenocopies *mulet*, indicating that TBCEL is required in the germline for individualization. RNAi driven by *bam-Gal4* has previously revealed cell-autonomous effects in the germline and has also been shown to markedly reduce the level of proteins required for individualization (Couderc et al., 2017). RNAi against TBCEL has also been shown to increase microtubule stability (Keller and Lauring, 2005; Bartolini et al., 2005). This, combined with our observations of persistent cytoplasmic microtubules in males undergoing RNAi (data not shown), indicates that reducing TBCEL levels in the germline would cause cytoplasmic microtubules to persist, thus phenocopying *mulet.*

Despite its success in rescuing that individualization phenotype of *mulet* mutant testes, the *tub-Gal4* driver was not able to phenocopy *mulet* in knockdown experiments. Conversely, TBCEL expression driven by *bam-Gal4-VP16* was incapable of rescuing the *mulet* mutant phenotype. Perhaps expression of TBCEL in spermatogonia did not produce enough protein necessary for rescuing post-meiotic defect. Perhaps *bam-Gal4-*driven transcription of hairpin *tbcel* RNA in spermatogonia initiated a chain reaction in which siRNAs acted as primers to initiate further synthesis of double-stranded *tbcel* RNA by an RNA-dependent RNA polymerase. Such an effect has been well documented (Yang et al., 2014; Tsai et al., 2015; Taochy et al., 2017). Less clear is the failure of *tub-Gal4* to drive observable knockdown of TBCEL. Perhaps the critical period for double-stranded RNA amplification is in spermatogonia, or perhaps the earlier spermatogonial expression driven by *bam-Gal4* allows more time for amplification of double-stranded RNA. In either case, these results confirm *bam-Gal4* as a driver of RNAi in the post-meiotic germline, and introduce *tub-Gal4* as a tool for rescuing such defects.

Consistent with increased Gal4 activity at elevated temperatures (Duffy, 2002), we consistently observed a more severe individualization phenotype in males undergoing RNAi at 28°C compared to more modest disruptions in males reared at 25°C. Consistent with the mild individualization phenotype of males homozygous for *mlt^G18151^*, a null allele of *mulet* (Fabrizio et al., 2012), we also observed a mild individualization phenotype in males undergoing severe RNAi at 28°C with augmented Dicer expression. These results were also confirmed by fertility testing, where we observed decreased fertility when RNAi was induced at 25°C, almost complete sterility when RNAi occurred at 28°C and improved fertility when RNAi experiments were conducted at 28°C with supplemental Dicer. Interestingly, RNAi-mediated knockdown of TBCEL at 28°C in the absence of Dicer and at 25°C in the presence of Dicer both resulted in almost complete sterility, suggesting equivalent knockdown levels. Thus, it appears that increasing the temperature to 28°C augments RNAi activity, to the same degree as excess Dicer at 25°C.

RNAi-mediated knockdown of TBCEL was confirmed using a polyclonal anti-TBCEL antibody. We are confident that the antibody is a reliable marker for TBCEL, as testes dissected from hemizygous males only reveal background staining as compared to wild type. This staining pattern was mirrored in knockdown experiments; testes dissected from control males exhibited prominent staining in elongated spermatid cysts, while testes dissected from knockdown males revealed background staining. While the polyclonal anti-TBCEL antibody was a reliable indicator of knockdown, it failed to discriminate among levels of RNAi. Indeed, testes dissected from males undergoing RNAi at 25°C in the absence of supplemental Dicer appeared to have the same background staining as testes undergoing RNAi at 28°C with enhanced Dicer activity. Despite these limitations, we are confident that RNAi was augmented by both increasing temperature from 25°C to 28°C and by the addition of *UAS-Dicer* based on the observed phenotypes. Males undergoing RNAi in the absence of supplemental Dicer at 28°C consistently exhibit more severely disrupted ICs and decreased fertility as compared to males of the same genotype raised at 25°C. Adding supplemental Dicer at 28°C consistently resulted in less severe IC disruptions and increased fertility; much like testes dissected from males homozygous for the *mlt^G18151^* null allele. Thus, since the phenotypes observed in knockdown experiments mirror previously documented phenotypes of known P-element insertions, we are certain that *tbcel* was reliably knocked down in using RNAi.

### Model for the role of microtubules in the various *mulet* mutant and knockdown phenotypes

**Fig. 6.**
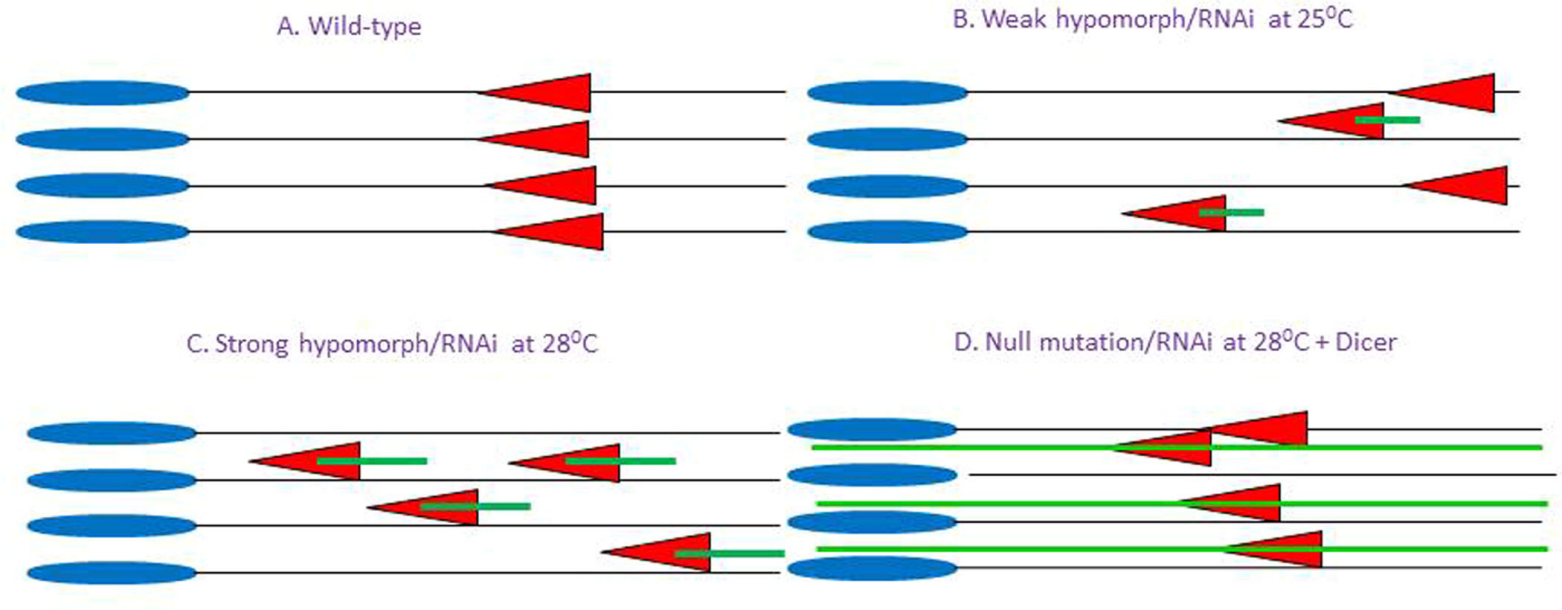
**Model for IC disruptions by cytoplasmic microtubules.** (A) Normal ICs in wild-type cysts, absent cytoplasmic microtubules. (B) Mild IC disruptions caused by persistence of a few microtubule fragments when TBCEL is moderately reduced. (C) Severe IC disruptions caused by persistence of many microtubule fragments when TBCEL is heavily reduced. (D) Almost normal ICs permitted by alternate microtubule tracks when TBCEL is absent.

These data support a model in which cytoplasmic microtubules must be removed by TBCEL prior to the departure of the IC from the spermatid nuclear bundle so that the IC will follow proper flagellar ‘tracks’ to the end of the sperm tails, resulting in successful individualization (Fig. 6A). When TBCEL levels are moderately reduced, such as in RNAi-mediated knockdown at 25°C, less TBCEL is produced, and thus some cytoplasmic microtubules would persist between the flagella, causing occasional ‘derailment’ of investment cones, which is precisely what we observe in ‘mildly disrupted ICs’ (Fig. 6B). The cones would follow these microtubule fragments to their termini, leaving the cones stranded and unable to reach flagellar tip. IC disruptions would worsen in strong hypomorphic alleles, and under conditions of strong RNAi, such as in the absence of Dicer at 28°C and the presence of Dicer at 25°C (Fig. 6C). In these cases, even less TBCEL would be produced, resulting in less degradation and thus more microtubule fragments between the flagella, leading to more cone derailments and severely disrupted ICs. Finally, this model explains why increased fertility and improved IC structure are observed in homozygous null mutant males and in males undergoing severe RNAi at 28°C augmented by supplemental Dicer. In these testes, very little or no TBCEL is produced, resulting in nearly intact cytoplasmic microtubules during individualization (Fig. 6D). Thus, when investment cones become derailed, since the microtubules are largely intact, the cones follow these microtubules to the end of the cysts, thus completing individualization.

### TBCEL's role in spermatogenesis may have evolved as a metazoan adaptation to a multicellular lifestyle

Unlike its paralog TBCE, TBCEL is not present in all eukaryotes, but rather is both present and highly conserved in all metazoans as one member of a group of 526 metazoan-specific genes known as the ‘metazoanome’ (Frédéric et al., 2013). Interestingly, many of these genes are expressed in the nervous system, and may have evolved in response to the demands of multicellularity. Perhaps a microtubule destabilizer was needed in addition to the microtubule-stabilizing activities of TBCE to coordinate the growth and shrinkage of microtubules in neuronal processes, thus allowing for remodeling of neural networks (Frédéric et al., 2013). Perhaps a similar balance is needed in spermatogenesis. Cytoplasmic microtubules must be assembled to allow for cystic elongation, and then degraded by TBCEL to allow for individualization. Thus sperm development, along with nervous system development, requires extensive microtubule dynamics and may also be a key factor in metazoan evolution. The fact that *mulet* has a neural and a spermatogenesis phenotype (Nuwal et al., 2012) supports this hypothesis. TBCEL is also preferentially expressed in human testes (Bartolini et al., 2005), suggesting that a similar balance between microtubule stabilization and destabilization is needed during human spermatogenesis.

## MATERIALS AND METHODS

### Fly husbandry

Flies were raised on JazzMix (226.8 g/1.2 l water) at 25°C. All crosses were conducted at 25°C, except in the RNAi experiments, where certain crosses were conducted at 28°C (see below). *P{ry+=A92}/mlt[1]/CyO; ry[506] [1]* (FBst0002491) was generated by insertion of *P{A92}* into the 5′-UTR of *mulet* and is a hypomorphic male-sterile allele (Fabrizio et al., 1998, 2012)*.* *y*^*1*^ *w*^*1*^; *P{EP}mlt[G18151**]* (FBst0027434) was generated by an insertion of *P{EP}* into the coding sequence of *mulet* and is a null allele of *mulet* (Celniker et al., 2002; Fabrizio et al., 2012) Both were obtained from the Bloomington Stock Center. *yw; P{w+y+=EPgy}CG12214[EY02157] (*FBal0162482), known simply as ‘*EP-CG12214*’, is an enhancer-promoter line obtained from Bloomington. Since the P{EPgy2}-insertion is within the 5′-UTR of *mulet*, *EP-CG1224* functions both as a hypomorphic mutant allele in the absence of a Gal4 driver (Fabrizio et al., 2012) and as a *UAS-CG12214* rescue construct in the presence of *tub-Gal4* (see below). *ms(2)4210* (FBal0090223), was generated in the lab of M. T. Fuller (Department of Developmental Biology, Stanford University) by insertion of *P{LacW}* into the 5′-UTR of *mulet* and is also a hypomorphic male-sterile allele (Fabrizio et al., 1998, 2012)*. w[1118]; Df(2R)BSC 281/CyO* (46F1; 47A9, 6,012,734…6,350,379) (FBst0023666), and *w[1118]; Df(2R)BSC350/CyO* (46F1;46F9, 2R:6,012,735…6,160,584) (FBst0024374) are deficiency stocks that uncover the *mlt* genomic region and were also obtained from the Bloomington Stock Center. *don juan-GFP (dj-GFP-88)* (Santel et al., 1997, 1998) was crossed into the *ms(2)4210* mutant background in order to generate an *ms(2)4210/CyO; dj-GFP* stock (Bazinet and Rollins, 2003).

*y[1] w[1118]; P{w[+mC]=UAS-mCD8::GFP.L}LL6, P{w[+mC]=tubP-GAL4}LL7/TM3, Sb[1]* (RRID:BDSC_30030), known simply as ‘*tub-Gal4*’ was obtained from the Bloomington Stock Center and used in all rescue experiments. *UAS-SMN-TBCEL* was a homozygous chromosome III stock obtained from the Buchner lab; the creation of this line is detailed below under ‘Generating the UAS-SMN-TBCEL vector and expressing it in flies.’ The *tub-Gal4* chromosome was crossed with *EP-CG12214* in order to create a *w; EP-CG12214/CyO; tub-Gal4/TM6Ubx* stock. Homozygous *CyO^+^* males carrying the *tub-Gal4* driver were analyzed for rescue and compared to homozygous *EP-CG12214* males without the driver (negative controls) and *EP-CG12214/CyO* heterozygous positive controls. In a separate set of experiments, this stock was also crossed with a *w; Df(2R)BSC 281/CyO; UAS-SMN-TBCEL/MKRS* stock (generated in our lab) to generate *w; EP-CG12214/Df(2R)BSC 281; tub-Gal4/UAS-SMN- TBCEL* males that were tested for rescue. CyO male offspring from this cross were used as a positive control, while *w; EP-CG12214/Df(2R) BSC281; UAS-′SMN-TBCEL* male offspring were used as negative controls. In the third rescue experiment, the *ms(2)4210* chromosome was also crossed into the *tub-Gal4* background in order to generate a *w; ms(2)4210/CyO; tub-Gal4/TM6Ubx* stock. This stock was also crossed to the *w; Df(2R)BSC 281/CyO; UAS-SMN-TBCEL/MKRS* stock, and *ms(2)4210/Df(2R)BSC281; tub-Gal4/UAS-SMN-TBCEL* males were tested for rescue. CyO male offspring from this cross were used as a positive control, while *w; ms(2)4210/Df(2R) BSC281; UAS-SMN-TBCEL* male offspring were used as negative controls. All rescue experiments were conducted at 25°C, and 0–1-day-old males were always used for dissection and fertility testing.

RNAi experiments were carried out using a chromosome II *UAS-CG12214 RNAi* (VDRC, 105480/KK) single insertion stock known to produce long hairpin double-stranded RNA (Dietzl et al., 2007) and a *bam-Gal4-VP16* driver generously provided by Josefa Steinhauer (Yeshiva University). RNAi was enhanced using a *P{UAS-Dcr-2.D}10* insertion on chromosome III obtained from the Bloomington Stock Center (RRID:BDSC_24651). RNAi against *CG12214* was generated simply by crossing *w; Sp/CyO; bam-Gal4-VP16* flies to *UAS-CG12214 RNAi/CyO* flies at either 25°C or 28°C. Testes from 0–1-day-old *UAS-CG12214 RNAi/Sp; bam-Gal4-VP16* or *UAS-CG12214 RNAi/CyO (Sp^−^); bam-Gal4-VP16* experimental males or *UAS-CG12214 RNAi* controls were dissected and processed as described below. The *w; CG12214 RNAi/CyO; UAS-Dicer/MKRS Sb* stock was also generated in our lab; crossing this stock to *w; Sp/CyO; bam-Gal4-VP16* produced w; *UAS-CG12214 RNAi; bam-Gal4-VP16/UAS-Dicer* males with enhanced RNAi that could be tested in parallel. For scoring purposes, testes from these knockdown testes were classified as either normal (indistinguishable from wild type) or exhibiting IC disruptions that were mild (disorganized investment cones are in close proximity thus the ICs almost looked intact), intermediate (ICs were obviously disrupted but ownership of the investment cones by a particular IC was clear) or severe (scattering was so severe that it was difficult to assign the scattered cones to a particular IC).

### Transmission electron microscopy

Testes from 0–1-day-old male flies (wild type or *mlt^1^/mlt^1^*) were dissected in 0.1 M phosphate buffer pH 7.4 and immediately placed in cold fixative (4% formaldehyde, 1% glutaraldehyde in 0.1 M KPO_4_ pH 7.4 overnight). Post-fixation, dehydration, infiltration and embedding were as described by Tokuyasu et al. (1972), except that Polybed 812/ Araldite was used. Thin sections were stained with uranyl acetate-lead citrate then observed with a transmission electron microscope (JEM-1200-EX, JEOL, Peabody, USA).

### Generation of anti-TBCEL antiserum

A His-tagged TBCEL protein was obtained by inserting the NotI-linked Tbcel cDNA of clone GH13040 (from BDGP Gold cDNA collection, primers for linker PCR: 5′-CATT GCGGCCGC ATG CCT TCC CTT TTG G-3′ and 5′-GATT GCGGCCGC TCA CTT CTT GGC ATC G-3′) in a pET 28a vector and transformation in *E. coli* BL21 cells. After purification by nickel-chelate affinity chromatography (following the supplier's protocol, Qiagen, Hilden, Germany) the His-TBCEL was injected into guinea pigs and antiserum was affinity purified as described earlier (Cordes and Krohne, 1993; Nieratschker et al., 2009).

### Generating the UAS-SMN-TBCEL vector and expressing it in flies

The NotI-linked Tbcel cDNA was inserted into NotI-linearized pMT-SMN1-30 vector (pMTagIt, Kroiss et al., 2009). The resulting pMT-SMN-Tbcel vector was linearized with XbaI and partially digested with KpnI, the resulting fragments were inserted in the XbaI- and KpnI- digested pUAST vector (Brand and Perrimon, 1993) to produce the pUAST-SMN1-30-Tbcel-cDNA vector which governs the expression of Survival of Motor Neuron (SMN)-tagged TBCEL under the control of the yeast UAS enhancer in *Drosophila*.

### Live imaging of testis squashes by phase contrast and fluorescence microscopy

Testes from 0–1-day-old y[1] w[1118]; P{w[+mC]=UAS-mCD8::GFP.L}LL6, P{w[+mC]=tubP-GAL4}LL7/TM3, Sb[1] (RRID:BDSC_30030) males were dissected in *Drosophila* Ringers solution or 1× PBS with the seminal vesicles and accessory glands still attached. Testes were transferred to a drop of Ringers or 1× PBS on a slide (using the seminal vesicles and accessory glands as ‘handles’ so as not to rupture the testes), and then a coverslip was gently laid over the preparation using forceps. Excess liquid was removed by touching a Kimwipe to the edge of the coverslip. Testes were then analyzed by phase-contrast microscopy to visualize cell types and fluorescence microscopy to visualize GFP expression using a Nikon Eclipse 80i epi-fluorescence microscope with a digital camera attachment.

### Immunofluorescence

Testes from 0–1 day-old males were crudely dissected in *Drosophila* Ringers or 1× PBS and transferred immediately to a tube of Ringers (or 1× PBS) on ice. Testes were then fixed in 4% formaldehyde in buffer B (16.7 mM KH_2_PO_4_/K_2_HPO_4_ pH 6.8, 75 mM KCl, 25 mM NaCl, 3.3 mM MgCl_2_). Following fixation, testes were rinsed three times in PTx (PBS+0.1% Triton X-100), washed in PTx for 30 min and blocked in Blocking solution for at least 1 h (0.01% NaAzide and 3% BSA in PTx). Tissues being processed for immunofluorescence were incubated with monoclonal anti-acetylated tubulin (1:300, Sigma-Aldrich, T6793), mouse anti-survival of motor neurons, or SMN (1:10, Immunoglobe, 0176-002) or guinea pig anti-TBCEL (1:50) in blocking solution on a rocker for 16 h at 4°C. Samples were then rinsed three times in PTx, washed with PTx (2×30 min) and incubated in with either a 1:64 dilution of anti-mouse IgG-FITC conjugate (Sigma-Aldrich, F2012) or Anti-Guinea Pig IgG FITC (Sigma-Aldrich, F6261) in blocking solution at room temperature for 1 h. Samples were rinsed and washed as above. Staining with rhodamine-conjugated phalloidin was done concurrently with secondary antibody staining in blocking solution (3 μg/ml). Testes were then rinsed three times and washed with PTx (2×30 min) and sometimes stained with 1 µg/ml Hoechst 33258 in PTx. After washing with PTx, testes were finely dissected from remaining carcasses in 50% glycerol and then mounted in 90% glycerol. Slides were observed using a Nikon Eclipse 80i epi-fluorescence microscope with a digital camera attachment or a Leica TCS SL confocal microscope (see below).

### Confocal microscopy

Confocal micrographs were obtained using a Leica TCS SL confocal microscope with Argon 458 nm 476 nm, 488 nm and 514 nm, Green HeNe 543 nm, and Red HeNe 633 nm laser lines, equipped for scanning in two fluorescence channels and a transmitted light channel simultaneously. The scanner was mounted on a Leica DM IRBE inverted microscope with a galvanometer-driven z-stage for rapid live imaging, with plan apochromat objectives through the full range of magnifications, 10×, 20×, 40×, 63× and 100× and Interference Contrast optics for all objectives >10×. For examining the sample before confocal scanning (e.g. assessing the quality of the preparation, finding a field that you want to image intensively using the scanner, etc.), there is a 50 W mercury illuminator and standard Leica fluorescence cubes for FITC, TRITC and DAPI/Hoechst dyes.

### Statistical analyses

Data from the first set of rescue experiments [rescue of *ms(2)4210/Df(2R)BSC281*] and the RNAi experiments were processed using the Cosine Similarity Test, where the numerator is A. B is the dot product of vectors A and B, and the denominator is the product of square root of lengths of vectors A and B. Here, two samples are regarded as vectors A and B in 4-dimentional Euclidian space:
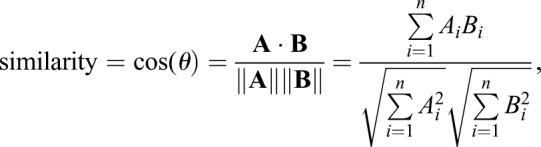
where *i* is an index that runs from 1 to *n* where *n* is the dimension of data set (for this data set *n* is 4). Data from the second and third set of rescue experiments (rescue of homozygous and hemizygous *CG12214[EY02157]* males were processed using the Euclidian Measure of Similarity:
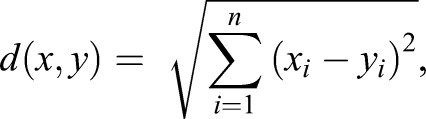
where *x* and *y* are points in Euclidean space (where like before *n* is equal to 4). Here we are considering each sample as a point in 4-dimentional Where Euclidean space. Then the Euclidian Measure of Similarity is defined as distance between two points denoted by *d*, which is defined as square root of sum of the square of differences of data points, component wise.

A Chi-Square test (calculated via Microsoft Excel) was employed for analysis of the RNAi data to determine whether or not a statistically significant relationship exists between groups.

## Supplementary Material

Supplementary information
