# Supplementary material for: Tubulin-binding cofactor E-like (TBCEL), the protein product of the mulet gene, is required in the germline for the regulation of inter-flagellar microtubule dynamics during spermatid individualization
Source: Biol Open. 2020 Feb 26;9(2):bio049080. doi: 10.1242/bio.049080 (PMC7055396; doi:10.1242/bio.049080)
Supplement: Supplementary information [file biolopen-9-049080-s1.pdf]

Supplemental Material

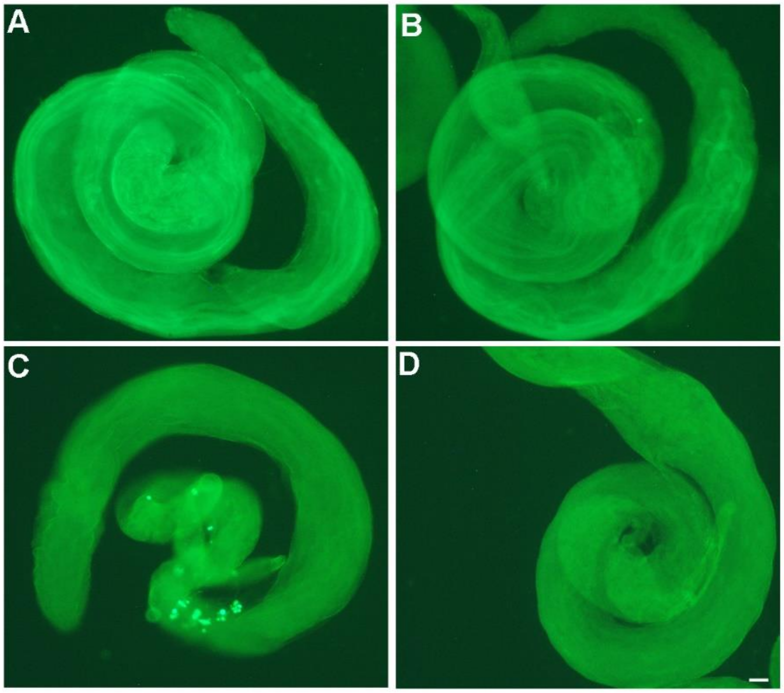

Figure S1. TBCEL is expressed in elongated spermatid cysts. Testes were probed with polyclonal guinea pig anti-TBCEL antibody (1:50) and detected with FITC-tagged anti-guinea pig 2^0 antibody (1:400). TBCEL is detected in wild-type testes (**A and B**) but not in testes from *CG12214 /Df(2R)BSC 281* (**C**) or *CG12214 / Df(2R)BSC350* (**D**), hemizygous males. Bar, 30µm

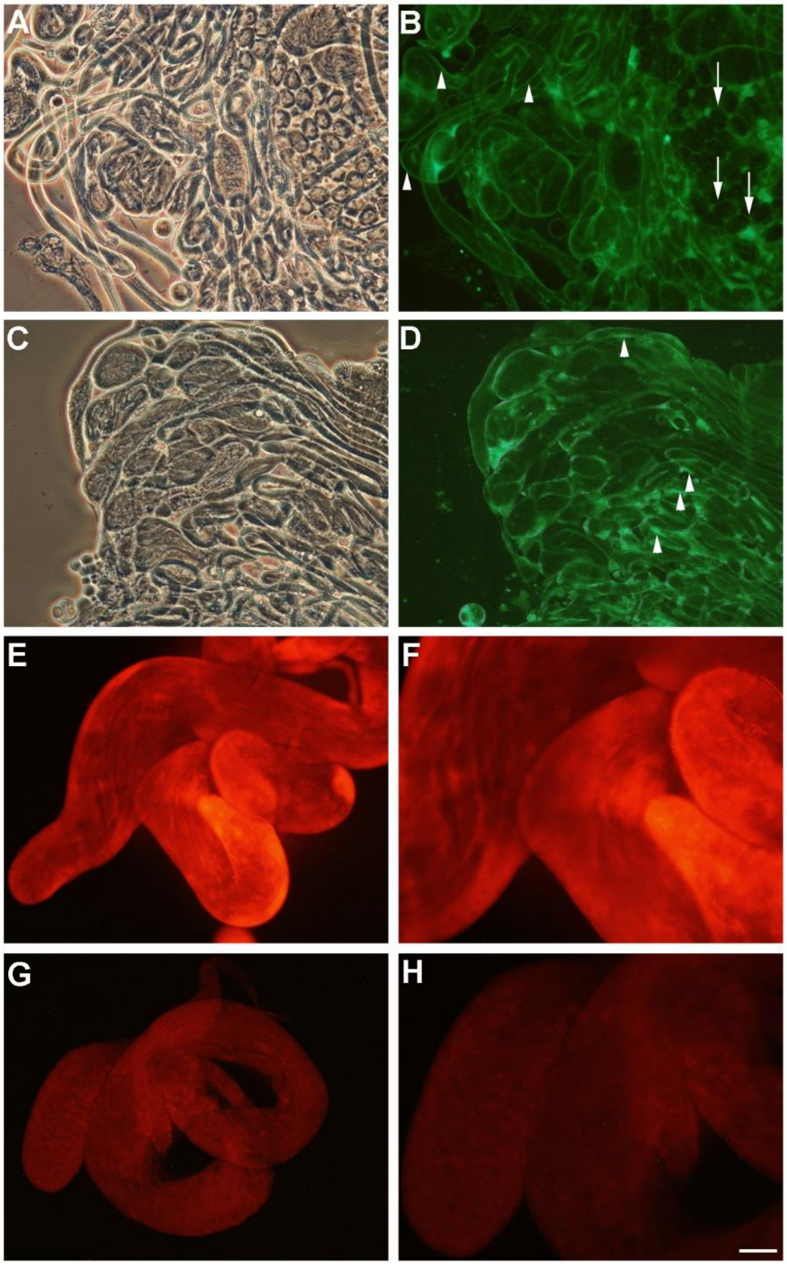

Figure S2. Tubulin-Gal 4 drives expression in post-meiotic spermatid cysts. (A-D) Live *tub-Gal4, UAS-mCD8-GFP* testes were analyzed for GFP expression. GFP expression in obvious in elongated cysts (*arrowheads* in B,D) and in primary spermatocytes (*arrows* in D). Phase contrast micrographs of **B** and **D** are shown in **A** and **C,** respectively. Low **(E,G)** and high **(F,H)** magnification images of testes from *UAS-SMN-TBCEL/Y; tub-Gal 4, UAS-mCD8-GFP/+* **(E,F)** and *UAS- SMN-TBCEL/Y; TM3Sb/+* controls **(G,H)** were stained with anti-SMN antibodies. SMN-TBCEL expression was significantly higher in the experimental group (**A,B**) than in the controls (**C,D**). Bar (A-D, F,H) 20µm; (E,G) 40µm

Biology Open • Supplementary information

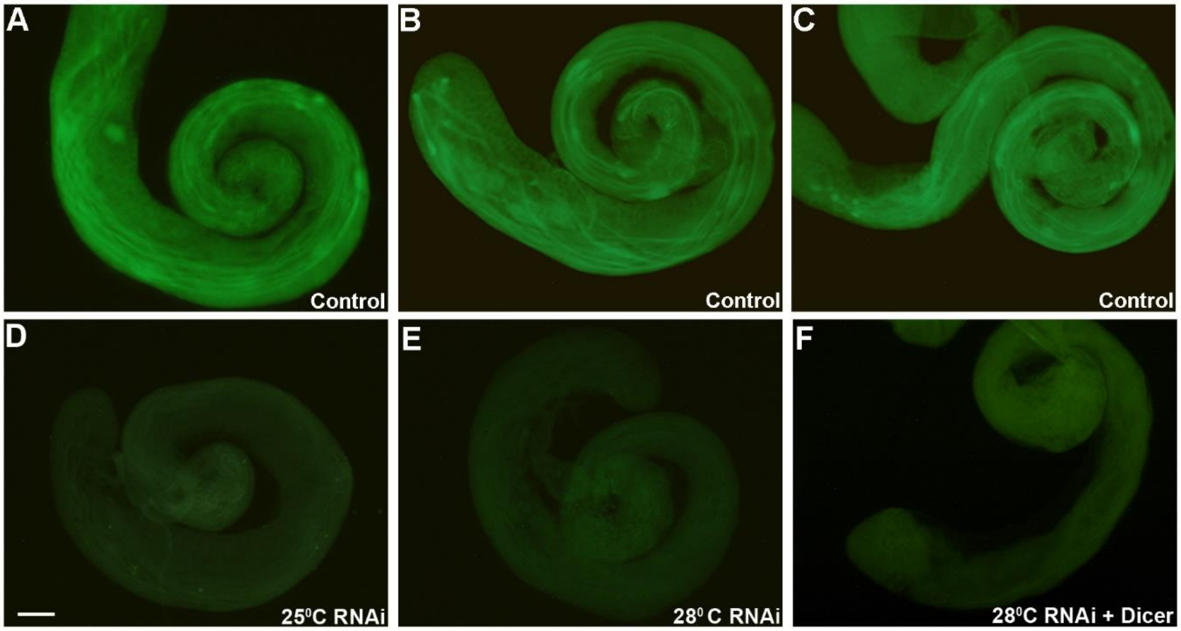

Figure S3: Germline specific *CG12214* RNAi reduces TBCEL levels. Control testes (UAS-*CG12214* RNAi alone) exhibited expression of TBCEL (**green**) in elongated cysts **(A-C)**, while *bam-Gal4*-VP16-mediated RNAi knockdown resulted in a marked decrease of TBCEL expression at 25^{0}C **(D),** 28^{0}C**(E)** and 28^{0}C in the presence of Dicer **(F)**. Bar, 40µm

**Table S1: Overexpression of TBCEL with *tub-Gal4* rescues the male-sterility of *mulet***				
Genotype	Sterile	Semi-sterile	Sub-fertile	Fertile
		(1-10 pupae/vial)	(11-50 pupae/vial)	(80-100 pupae/vial)
EP-CG12214/EP-CG12214; tub-Gal4 (Experimental, 11 males tested)	1	4	6	0
EP-CG12214/CyO; tub-Gal4 (Positive Control, 10 males tested)	0	0	4	6
EP-CG12214/EP-CG12214 (Negative control, not viable)	N/A	N/A	N/A	N/A
EP-CG12214/Df(2R)BSC281; tub-Gal4 (Experimental, 14 males tested)	9	3	2	0
EP-CG12214 or Df(2R)BSC281/CyO; tub-Gal4 (Positive Control, 10 males tested)	3	0	7	0
EP-CG12214/Df(2R)BSC281 (9 males tested)	9	0	0	0

Biology Open • Supplementary information

Biology Open (2020): doi:10.1242/bio.049080: Supplementary information

**Table S2 : Fertility Testing of TBCEL Knockdown Males**					
Genotpe	Temperature	Sterile	Semi-sterile	Subfertile	Fertile
	(Degrees Celsius)	(1-10 pupae/vial)	(11-50 pupae/vial)	(11-50 pupae/vial)	(80-100 pupae/vial)
UAS-TBCEL RNAi alone	25	2	0	0	9
UAS-TBCEL RNAi; bam-Gal4VP16	25	0	6	3	0
UAS-TBCEL RNAi; bam-Gal4VP16/UAS-Dicer	25	8	2	0	0
UAS-TBCEL RNAi alone	28	1	0	0	7
UAS-TBCEL RNAi; bam-Gal4VP16	28	5	2	0	0
UAS-TBCEL RNAi; bam-Gal4VP16/UAS-Dicer	28	1	6	2	0

Biology Open • Supplementary information
